# Hexokinase 2 controls cellular stress response through localization of an RNA-binding protein

**DOI:** 10.1038/cddis.2015.209

**Published:** 2015-08-06

**Authors:** L Courteau, J Crasto, G Hassanzadeh, S D Baird, J Hodgins, U Liwak-Muir, G Fung, H Luo, D F Stojdl, R A Screaton, M Holcik

**Affiliations:** 1 Molecular Biomedicine Program, Children's Hospital of Eastern Ontario Research Institute, 401 Smyth Road, Ottawa, Ontario K1H 8L1, Canada; 2 Department of Biochemistry, Microbiology and Immunology, University of Ottawa, 451 Smyth Road, Ottawa, Ontario K1H 8M5, Canada; 3 Department of Pathology and Laboratory Medicine, James Hogg Research Center, University of British Columbia, Vancouver, British Columbia V6Z 1Y6, Canada; 4 Department of Pediatrics, University of Ottawa, 451 Smyth Road, Ottawa, Ontario K1H 8M5, Canada

## Abstract

Subcellular localization of RNA-binding proteins is a key determinant of their ability to control RNA metabolism and cellular stress response. Using an RNAi-based kinome-wide screen, we identified hexokinase 2 (HK2) as a regulator of the cytoplasmic accumulation of hnRNP A1 in response to hypertonic stress and human rhinovirus infection (HRV). We show that inhibition of HK2 expression or pharmacological inhibition of HK2 activity blocks the cytoplasmic accumulation of heterogeneous nuclear ribonucleoprotein A1 (hnRNP A1), restores expression of B-cell lymphoma-extra large (Bcl-xL), and protects cells against hypertonic stress-induced apoptosis. Reduction of HK2 protein levels by knockdown results in decreased HRV replication, a delay in HRV-induced cell death, and a reduced number of infected cells, all of which can be rescued by forced expression of a cytoplasm-restricted hnRNP A1. Our data elucidate a novel role for HK2 in cellular stress response and viral infection that could be exploited for therapeutic intervention.

Regulation of protein expression through RNA metabolism is a key aspect of cellular homeostasis. Upon specific cellular stresses, distinct transcripts are selectively controlled to modify protein output in order to quickly and appropriately respond to stress.^1^ This is accomplished through a large assortment of specialized RNA-binding proteins (RBPs) that control diverse aspects of RNA metabolism ranging from mRNA processing to export, translation, and degradation. Frequently, the same RBP can exhibit multiple roles that are dependent on its subcellular localization within the cell, suggesting that the diverse roles of RBPs in mRNA metabolism are controlled, at least in part, by the compartmentalization of these proteins.^2, 3, 4^ Although there are sporadic reports that investigated the mechanism(s) that control localization of RBPs, a systematic approach to identify factors and pathways involved in this control has not been undertaken.

Heterogeneous nuclear ribonucleoprotein A1 (hnRNP A1) is a highly conserved RBP that plays diverse roles in RNA metabolism, including telomere repair, alternative mRNA splicing, mRNA export, stress granule formation, miRNA processing, and selective mRNA translation.^5^ HnRNP A1 was shown to contain a nuclear localization signal, called M9, that enables both its nuclear entry and exit.^6^ HnRNP A1 normally shuttles between the nucleus and the cytoplasm, with the bulk of the protein displaying nuclear localization.^7^ During cellular stress such as hypertonic shock, however, hnRNP A1 undergoes phosphorylation at several serine residues at the C-terminus (known as F-peptide, adjacent to the M9 sequence) that blocks its transportin-dependent nuclear import.^8^ Using chemical inhibitors, it was shown that the p38 mitogen-activated protein kinase (MAPK) pathway is partially responsible for this phosphorylation.^8^ Interestingly, the accumulation of hnRNP A1 in the cytoplasm has different consequences for distinct mRNAs. For example, although the cytoplasmic hnRNP A1 destabilizes mRNA of cIAP1 in UV-irradiated cells^9^ and suppresses internal ribosome entry site (IRES)-mediated translation of X chromosome-linked IAP (XIAP) and B-cell lymphoma-extra large (Bcl-xL) during hypertonic shock,^10, 11^ the same cytoplasmic accumulation drives translation of human rhinovirus (HRV) RNA and is required for efficient infection.^12^ It was shown recently that hnRNP A1 is also a substrate of S6K2 kinase downstream of fibroblast growth factor-2 (FGF-2) signaling whereby S6K2 phosphorylates hnRNP A1 on a site (Serine 4) distinct from the F9 motif and promotes nuclear export of specific mRNAs.^13^ In addition, a novel link was suggested between eukaryotic initiation factor 2 *α* subunit (eIF2*α*) phosphorylation and subcellular localization of hnRNP A1 during hypertonic stress, although the mechanism of this relationship is not yet known.^10^ Collectively, these data suggest that there exist multiple signaling pathways controlling hnRNP A1 localization.

In this work, we set out to determine the signaling molecules that regulate localization of hnRNP A1, and the biological consequences of this regulation. We screened a library of siRNA pools targeting the kinome subset of the human genome and identified several candidate kinases that regulate cytoplasmic accumulation of hnRNP A1 in response to hypertonic stress. We further characterized one such kinase, hexokinase 2 (HK2), and showed that inhibition of HK2 expression or activity blocks the cytoplasmic accumulation of hnRNP A1, restores expression of Bcl-xL, and consequently protects cells against hypertonic stress-induced apoptosis. Furthermore, we demonstrated that during HRV infection, knockdown of HK2 levels by siRNA results in decreased HRV replication, a delay in HRV-induced cell death, and reduced number of infected cells. Importantly, transient transfection of cells with the cytoplasm-restricted hnRNP A1, but not the nucleus-restricted variant of hnRNP A1, restores HRV infection in cells with reduced levels of HK2. These data elucidate a novel role for HK2 in rhinovirus infection that could be exploited for antiviral intervention.

## Results

### Distinct kinases control cytoplasmic accumulation of hnRNP A1 during hypertonic shock

**Figure 1:**
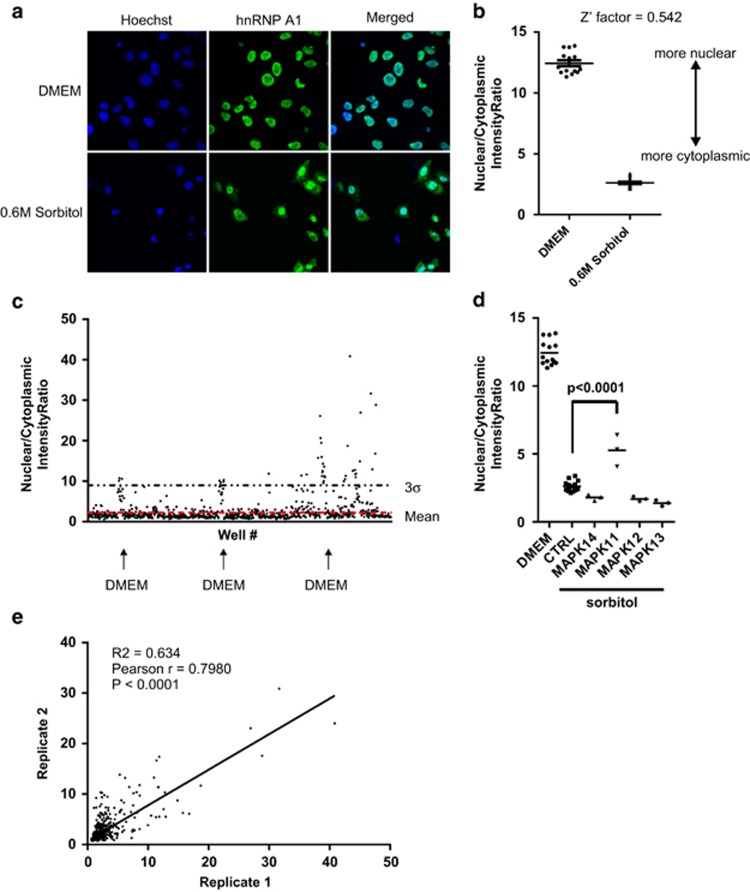
RNAi screen identifies several candidate kinases that regulate cytoplasmic accumulation of hnRNP A1 in response to hypertonic stress. (**a**) U2OS cells were treated with 0.6 M sorbitol for 4 h and the subcellular localization of hnRNP A1 was determined by immunofluorescence. Nuclei were visualized with Hoechst staining. (**b**) The nuclear/cytoplasmic intensity ratio of hnRNP A1 distribution in experiment (**a**) was determined as described in the Materials and Methods and the robustness of the assay was determined by the Z'-factor as previously described.^32^ (**c**) U2OS cells were reverse transfected for 72 h with a library of siRNA pools against 691 human kinases and kinase-related genes (the kinome subset of the Qiagen Human Druggable Genome siRNA Set Version 2.0) and subsequently treated with 0.6 M sorbitol for 4 h. The nuclear/cytoplasmic intensity ratio of hnRNP A1 was determined as in (**b**) and plotted. Each dot represents an siRNA pool. The red line represents the mean of nuclear/cytoplasmic intensity ratios across all plates. The dotted black line represents 3*σ*. Clusters of wells treated with DMEM instead of sorbitol are indicated with black arrows. Results from one replicate are shown. (**d**) The nuclear/cytoplasmic intensity ratio of hnRNP A1 for siRNA pools targeting the four members of the p38 MAPK family (MAPK14, MAPK11, MAPK12, and MAPK13) from all three replicates is shown. (**e**) Correlation plot between two replicates of the RNAi screen. Each dot represents an siRNA pool

**Figure 2:**
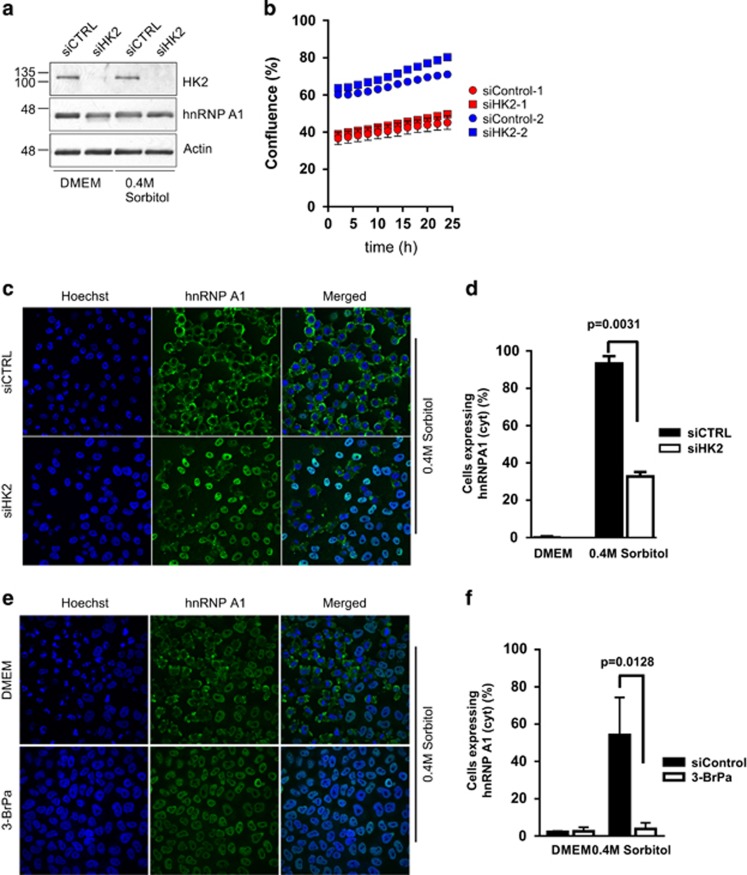
HK2 is required for cytoplasmic accumulation of hnRNP A1 during hypertonic stress. (**a**) HeLa T4+ cells were transfected with HK2-targeting (siHK2) or nontargeting control (siCTRL) siRNAs for 48 h and cell lysates were analyzed by western blot analysis. MW marker is indicated on the left. (**b**) HeLa T4+ cells transfected as in (**a**) were seeded at two different cell densities in 96-well format plates and cell confluency was monitored for 24 h by IncuCyte live cell imaging. (**c**) Representative confocal microscopy images of hnRNP A1 localization by immunofluorescence in sorbitol-treated cells transfected with HK2-targeting or nontargeting control siRNA. (**d**) Quantification of (**b**), where ∼250 cells per condition were counted; *P*=0.0031. (**e**) Representative confocal microscopy images of hnRNP A1 localization by immunofluorescence in sorbitol-treated cells previously treated with the HK2 inhibitor 3-BrPa. (**f**) Quantification of (**d**), where ∼2000 cells per condition were counted; *P*=0.0128

HnRNP A1 normally shuttles between the nucleus and the cytoplasm, with the bulk of the protein displaying nuclear localization.^7^ However, exposure of cells to various cellular stresses results in the accumulation of hnRNPA1 in the cytoplasm.^8, 10, 14^ Although several reports implicated a role for p38 MAPK signaling pathway in the control of hnRNP A1 localization in response to hypertonic stress, the systematic interrogation of signaling pathways has never been conducted. We therefore tested a library of siRNA pools against 691 human kinases and kinase-related genes (the kinome subset of the Qiagen Human Druggable Genome siRNA Set Version 2.0, Qiagen Inc., Germantown, MD, USA) for their effect on the cytoplasmic accumulation of hnRNP A1 in response to hypertonic stress. Treatment of U2OS cells with 0.6 M sorbitol resulted in a significant accumulation of hnRNP A1 in the cytoplasm (Figure 1a). In order to provide an unbiased scoring of the hnRNP A1 localization, we used an Acapella Image Analysis Software (Perkin Elmer, Waltham, MA, USA) script that calculated the nuclear/cytoplasmic intensity ratio of hnRNP A1 (see Materials and Methods). The treatment of cells with sorbitol resulted in a robust difference in the nuclear/cytoplasmic intensity ratio, with an excellent Z'-factor (Figure 1b). For the kinome screen, the cells were reverse transfected with siRNA pools and treated either with 0.6 M sorbitol or DMEM as a control. We observed that although the majority of siRNA pools had no appreciable effect on the nuclear/cytoplasmic intensity ratio of hnRNP A1, there were several pools that considerably attenuated cytoplasmic accumulation of hnRNP A1 (Figure 1c). Interestingly, knockdown of one isoform of p38 MAPK, MAPK11, but not the other three isoforms, reduced the cytoplasmic accumulation of hnRNP A1 (Figure 1d), in concordance with published reports that pharmacological inhibition of the p38 MAPK pathway blunts the cytoplasmic accumulation of hnRNP A1 during hypertonic shock.^8^ The quality of the screen was confirmed by the reproducibility of the data in two out of three biological replicates (Figure 1e). In order to confirm the data from the kinome screen, we selected one hit (HK2; nuclear/cytoplasmic intensity ratio=9.71) for further validation. Gene silencing of HK2 by an siRNA that is different from the siRNA pool used in the screen had no adverse effect on cell growth (Figure 2b) but resulted in the loss of cytoplasmic accumulation of hnRNP A1 in sorbitol-treated U2OS and HeLa cells (Figures 2c and d, Supplementary Figure S1, and data not shown). Similarly, treatment of HeLa cells with 3-bromopyruvate (3-BrPa), a specific HK2 inhibitor, before sorbitol treatment significantly blocked cytoplasmic accumulation of hnRNP A1 (Figures 2e and f). Overexpression of HK2 alone had no appreciable effect on the abundance of hnRNP A1 or its cytoplasmic accumulation, suggesting that HK2 is necessary but not sufficient for hnRNP A1 localization, or that an external stimulus (such as osmotic shock) is required for the activation of HK2–hnRNP A1 pathway (Supplementary Figure S2). These results demonstrate that HK2 is a *bona fide* regulator of hnRNP A1 localization during hypertonic stress.

### Inhibition of HK2 restores expression of Bcl-xL and protects cells from hypertonic stress-induced apoptosis

**Figure 3:**
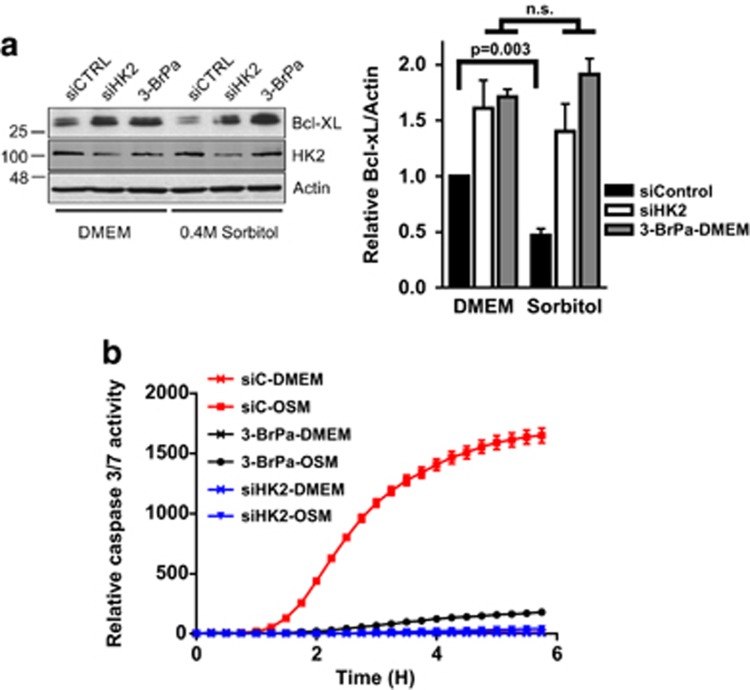
Knockdown or inhibition of HK2 restores expression of Bcl-xL and prevents hypertonic stress-induced cell death. (**a**) HeLa T4+ cells were transfected with HK2-targeting (siHK2) or nontargeting control (siCTRL) siRNAs, or treated with 3-BrPa and subsequently treated with 0.4 M sorbitol. Expression of Bcl-xL, HK2, and actin (loading control) was determined by western blot analysis (left). Bcl-xL expression relative to actin was quantified by densitometry (right). MW marker is indicated on the left. (**b**) Cells were treated as in (**a**) and caspase 3 and 7 activity was determined using the CellPlayer Kinetic Caspase-3/7 reagent and the IncuCyte Zoom. The y axis represents the number of fluorescent cells per image normalized to cell confluence; *n*=3 (3-BrPa treatment) and *n*=4 (siRNA treatment)

We have shown previously that hypertonic stress-induced cytoplasmic accumulation of hnRNP A1 results in the repression of Bcl-xL expression by blocking the IRES-mediated Bcl-xL translation, and subsequent cell death.^10^ We therefore tested whether inhibiting HK2 activity would affect Bcl-xL expression and cell survival in sorbitol-treated cells. HeLa cells were transfected with siHK2 or nontargeting siCTRL, or pretreated with 3-BrPa, and subsequently exposed to 0.4 M sorbitol for 2 h, and the expression of Bcl-xL was examined by western blot analysis (Figure 3a). We observed that sorbitol treatment reduced Bcl-xL expression as previously reported.^10^ We also showed that either knockdown of HK2 by siRNA or inhibiting the activity of HK2 by 3-BrPa before sorbitol treatment resulted in enhanced Bcl-xL expression. Furthermore, when we measured the activation of caspases 3 and 7 in sorbitol-treated cells, we observed complete inhibition of caspase activation in cells with reduced levels or activity of HK2 (Figure 3b). These results suggest a role for HK2 in regulating Bcl-xL expression and apoptosis, likely through modulating the subcellular localization of hnRNP A1.

### HK2 is required for rhinovirus-induced cytoplasmic accumulation of hnRNP A1

**Figure 4:**
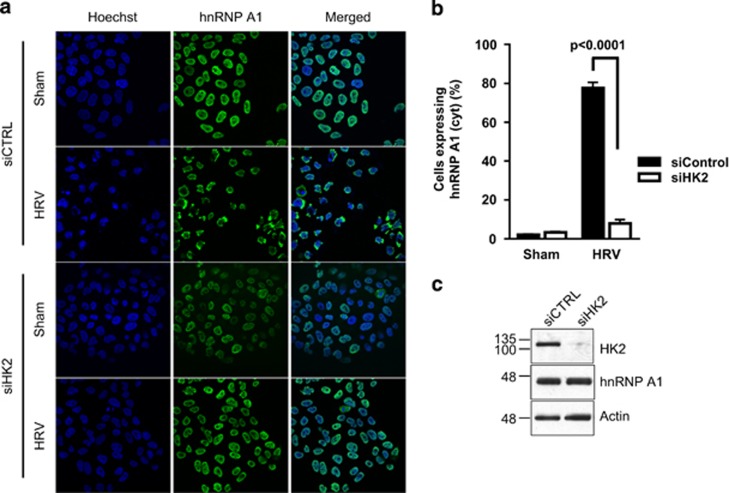
HRV-induced cytoplasmic accumulation of hnRNP A1 is mediated by HK2. (**a**) HeLa T4+ cells were transfected with HK2-targeting (siHK2) or nontargeting control (siCTRL) siRNA and subsequently infected with HRV (or PBS as a control) for 10 h and subcellular localization of hnRNP A1 was determined by immunofluorescence; nuclei were visualized by Hoechst staining. Representative immunofluorescence confocal images are shown. (**b**) Quantification of (**a**), where ∼250 cells per condition were counted; *P*=0.0001. (**c**) The efficiency of knockdown was confirmed by western blotting

**Figure 5:**
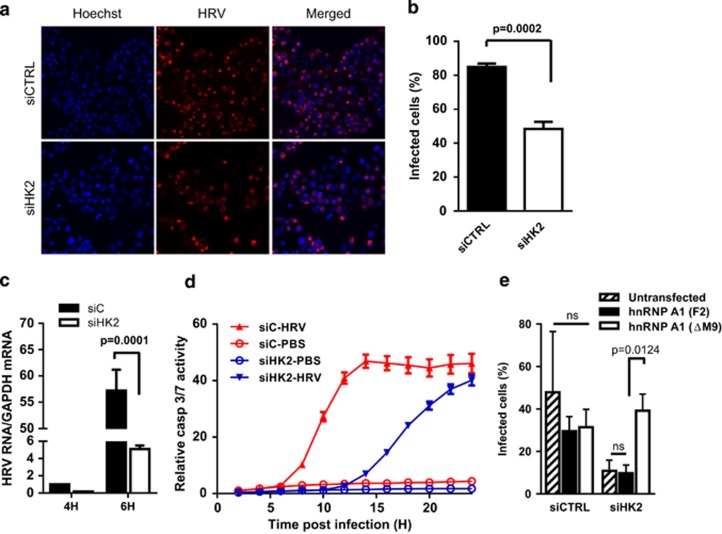
HK2 knockdown attenuates human rhinovirus infection that is rescued by forced expression of cytoplasm-restricted hnRNP A1. (**a**) HeLa T4+ cells were transfected with HK2-targeting (siHK2) or nontargeting control (siCTRL) siRNA for 48 h and subsequently infected with HRV for 10 h. The expression of viral capsid proteins was detected by immunofluorescence and nuclei were stained with Hoechst. Representative immunofluorescence confocal images are shown. (**b**) Quantification of the number of infected cells (identified as those that stained positive for viral capsid protein relative to the total number of cells identified by Hoechst) from (**a**), where ∼350 cells per condition were counted; *n*=3, *P*=0.0002. (**c**) Levels of HRV RNA in HK2-targeting (siHK2) or nontargeting control (siCTRL) siRNA-transfected HeLa T4+ cells infected for 4 or 6 h were determined by RT-qPCR. Absolute amount of HRV RNA normalized to GAPDH mRNA levels is shown; *n*=3, *P*=0001. (**d**) Kinetic analysis of caspase 3/7 activity in HRV-infected HeLa T4+ cells previously transfected with HK2-targeting (siHK2) or nontargeting control (siCTRL) siRNA. The y axis represents the number of fluorescent cells per image normalized to cell confluency; *n*=3, *P*<0.0001 for siHK2 *versus* siCTRL HRV-infected cells. (**e**) HeLa T4+ cells were transfected with HK2-targeting (siHK2) or nontargeting control (siCTRL) siRNA, subsequently transfected with plasmids expressing FLAG-hnRNP A1 F2 or FLAG-hnRNP A1ΔM9 for 24 h and then infected with HRV. The number of infected cells was determined as in (**a**), where ∼350 cells per condition were counted; *P*=0.0124

It has been shown that infection of cells with HRV leads to cytoplasmic accumulation of hnRNP A1,^15^ and subsequent enhancement of HRV IRES activity.^12^ We therefore tested whether inhibition of HK2 would impinge upon these processes as well. HeLa cells were transfected with siHK2 or nontargeting siCTRL followed by infection with HRV, and the hnRNP A1 localization was determined by immunofluorescence microscopy. We observed that infection of cells with HRV led to cytoplasmic accumulation of hnRNP A1 that was attenuated in siHK2-transfected cells (Figure 4). Furthermore, knockdown of HK2 resulted in a significantly reduced number of HRV-infected cells (Figures 5a and b), a significant decrease in HRV RNA accumulation (Figure 5c), and a delay in HRV-mediated apoptosis (Figure 5d). To determine whether the effect of HK2 on viral infection was specifically due to changes in hnRNP A1 localization, we transfected cells with plasmids expressing either the nucleus- (F2)^8^ or the cytoplasm-restricted (ΔM9)^16^ mutants of hnRNP A1. We observed that forced expression of cytoplasmic but not nuclear hnRNP A1 in cells with reduced levels of HK2 restored infection of HRV (Figure 5e). Taken together, these results demonstrate that cytoplasmic accumulation of hnRNP A1 is regulated, at least in part, by HK2 and is required for effective HRV infection.

## Discussion

The cellular localization of hnRNP A1 is altered during certain cell stresses, allowing hnRNP A1 to control selective translation of distinct mRNAs. Here, we used an RNAi-based kinome-wide screen and identified HK2 as a novel kinase that regulates cytoplasmic accumulation of hnRNP A1 during hypertonic stress and rhinovirus infection. Notably, siRNA-mediated knockdown of related hexokinases 1, 3, and 4 did not alter the cytoplasmic accumulation of hnRNP A1 during hypertonic stress (nuclear/cytoplasmic ratios of 1.449, 1.627, and 1.945, respectively, data not shown), suggesting that HK2 is unique among the hexokinases in regulating subcellular localization of hnRNP A1. This was further confirmed using the small-molecule inhibitor of HK2, 3-BrPa, as treatment of cells with 3-BrPa before hypertonic shock completely blocked accumulation of hnRNP A1 in the cytoplasm. Previous work identified the p38 MAPK pathway as being responsible for cytoplasmic accumulation of hnRNP A1 during hypertonic stress.^8^ Indeed, our kinome screen confirmed involvement of MAPK11 (p38 *β*), but not the other three p38 MAPK isoforms (Figure 1). These data demonstrate the advantage of a genetic approach to dissect complex signaling pathways over the use of chemical inhibitors that frequently lack target specificity.^17^

Our results raise an important question. How does HK2 regulate hnRNP A1 localization? As the known mechanism responsible for cytoplasmic accumulation of hnRNP A1 is phosphorylation within its C-terminal F peptide motif, does HK2 phosphorylate hnRNP A1 directly, or does it act through another signaling pathway? HK2 is one of the key enzymes of glucose metabolism and its role is to phosphorylate glucose in order to initiate glycolysis. Expression of HK2 is frequently dysregulated in cancer, contributing to the Warburg effect.^18^ Although the traditional role of HK2 is in glucose metabolism, recent evidence implicates a role for HK2 in the control of other pathways. Proteome-wide interaction studies identified new interaction partners of HK2, melanoma antigen family A 12 (MAGEA12) and ubiquilin 1 (UBQLN1), although the biological implications of these interactions are not known.^19, 20^ Recently, HK2 was shown to physically interact with TORC1 through the mechanistic target of rapamycin (mTOR) signaling motif (TOS), resulting in decreased mTORC1 activity, although this interaction does not result in the phosphorylation of TORC1.^21^ Interestingly, the TOS motif is found in HK2 but not in the other members of the hexokinase family. In our experiments, we did not observe phosphorylation of hnRNP A1 by recombinant HK2 (data not shown), suggesting that HK2 either potentiates the activity of another hnRNP A1 kinase or enables hnRNP A1 phosphorylation by physical association with hnRNP A1. Identification of HK2 adds to the number of signaling pathways that regulate cytoplasmic accumulation of hnRNP A1 and suggests a crosstalk of distinct pathways in response to stress. Our screening approach may not have been sensitive enough to uncover the hierarchical organization of these pathways; however, the fact that siRNA-mediated knockdown of either HK2 or MAPK11 (and possibly other kinases) is sufficient to attenuate the cytoplasmic accumulation of hnRNP A1 suggests that these pathways genetically interact.

The effect of the cytoplasmic accumulation of hnRNP A1 on selective translation has been previously explored; of note, the mRNA targets of hnRNP A1, such as Bcl-xL, XIAP, Apaf-1, FGF-2, or c-myc, are all translated by an IRES-dependent mechanism under the conditions of cellular stress. HnRNP A1 was proposed to be one of the key IRES *trans*-acting factors that regulates cellular stress response via selective translation.^5, 10^ We observed that both the inhibition of HK2 activity by 3-BrPa and inhibition of HK2 expression blocked hypertonic stress-induced apoptosis by allowing translation of Bcl-xL that is otherwise repressed by cytoplasmic hnRNP A1. Together, these findings strongly support the previously proposed view that cytoplasmic accumulation of hnRNP A1 during hypertonic stress is the critical factor that shifts the balance from survival to apoptosis.^10^

HnRNPA1 is also associated with viral infection. In particular, hnRNP A1 was shown to interact with the 5′ untranslated regions of human rhinovirus, enterovirus 71 and Sindbis virus, and promote viral replication and translation.^22, 23^ Importantly, cytoplasmic accumulation of hnRNP A1 during rhinovirus infection is critical for successful infection as cytoplasmic hnRNP A1 promotes IRES-dependent translation of viral RNA.^12^ Many nuclear-localized proteins accumulate in the cytoplasm following enteroviral infection.^15, 24^ This is believed to be because of the inhibition of the nuclear import by cleaving the components of the nuclear core complex, in particular Nup153 and p62. However, the nuclear–cytoplasmic shuttling of hnRNP A1 was shown to be mediated by transportins,^25^ suggesting that the cytoplasmic accumulation of hnRNP A1 during rhinovirus infection could be mediated by a different mechanism from other RBPs. Our data support this model as the inhibition of HK2 and the resultant prevention of accumulation of hnRNP A1 in the cytoplasm attenuated rhinovirus infection. Importantly, forced expression of cytoplasm-restricted hnRNP A1 fully restored HRV infection, confirming that the attenuated viral infection in HK2-depleted cells was because of the absence of hnRNP A1 in the cytoplasm. As other enteroviruses also rely on hnRNP A1 for effective replication, we tested whether blocking cytoplasmic accumulation of hnRNP A1 would result in a similar attenuation of their infection. However, siRNA-mediated knockdown of HK2 had no impact on coxsackievirus B3-induced cytoplasmic localization of hnRNP A1 (Supplementary Figure S3A, left panel). The number of infected cells and the level of viral protein were also found to be unchanged following gene silencing of HK2 (Supplementary Figure S3A, right panel, and B). Several other ITAFs used by rhinovirus, such as PCBP2 and PTB, are cleaved by rhinovirus encoded proteases;^26^ this is, however, not observed with hnRNP A1 in either the rhinovirus- or the coxsackievirus-infected cells (data not shown and Supplementary Figure S3B). Together, these data suggest that the mechanism of regulation of hnRNP A1 differs from that of other cellular RBPs that have functions in viral translation.

The conclusions from this study may have broad applications for therapeutic approaches to cancer and viral infection. Deletion of HK2 has no apparent deleterious effect on normal tissues in mice,^27^ suggesting that HK2 inhibition could be used to treat HRV infection (common cold), for which there is currently no effective therapy. In contrast, hnRNP A1 levels and its cytoplasmic accumulation are increased in numerous cancers, and hnRNP A1 is a direct target of an anticancer drug quercetin.^28^ HK2 is overexpressed in cancer cells, is a major contributor to the Warburg effect, and specific targeting of HK2 shows therapeutic promise for the treatment of cancer.^27^ Our data propose a novel link whereby HK2 activity contributes to the cytoplasmic accumulation of hnRNP A1. Intriguingly, this could explain the oncolytic properties of some enteroviruses, such as poliovirus, senecavirus, and ECHO virus (commercially available as Rigvir),^29^ as their replication would be more effective in cells with an excess of HK2. If true, combining HK2 inhibitors with oncolytic viruses could inadvertently limit their therapeutic utility for the treatment of cancer.

## Materials and Methods

### Cell culture, reagents, and transfection

HeLa T4+ and U2OS cells were cultured in HyClone high-glucose Dulbecco's modified Eagle's medium (DMEM, Thermo Scientific, Waltham, MA, USA) supplemented with heat-inactivated 10% fetal calf serum, 2 mM L-glutamine, and 1% antibiotics (100 units/ml penicillin–streptomycin) at 37 °C and 5% CO_2_. For knockdown experiments, 5.0E04 cells were seeded in a 12-well plate in DMEM containing no antibiotics and grown for 24 h. Cells were then transfected with 20 nM nontargeting siRNA (negative control, GE Healthcare Dharmacon Inc., Mississauga, ON, Canada) or siHK2 (Silencer Select siRNA, Ambion Life Technologies Inc., Burlinton, ON, Canada) using Lipofectamine RNAiMAX transfection reagent (Invitrogen, Burlington, ON, Canada) following the manufacturer's protocol for forward transfection, and then grown for 48 h. Alternatively, cells were transfected with 1.5 *μ*g plasmid DNA using Lipofectamine 2000 transfection reagent (Invitrogen) following the manufacturer's protocol. The hnRNP A1 F2 and ΔM9 expression plasmids were generously provided by Dr. Stephen Lewis (Moncton, NB, Canada) and by Dr. Maria Hatzoglou (Cleveland, OH, USA), respectively; both have been previously described.^8, 16^ The FLAG-HK2 expression plasmid was generated by PCR amplification of HK2 coding sequence from GFP-HK2 construct (Addgene plasmid 21920, Cambridge, MA, USA)^30^ and incorporating the FLAG epitope into the pcDNA3.1 expression vector by PCR as previously described.^11^ HK2 inhibitor 3-BrPA (Calbiochem, Etobicoke, ON, Canada) was reconstituted in sterile ddH_2_O and was added directly to cells in culture medium at desired time at a concentration of 100 *μ*M.

### RNAi kinome screen

An arrayed library of siRNA pools (4 siRNAs per pool) was used to target 691 genes annotated as being relevant to kinase activity (the kinome subset of the Qiagen Human Druggable Genome siRNA Set Version 2.0, Qiagen Inc.). U2OS cells were seeded at 1000 cells/well in 384-well plates in DMEM. Each plate included additional control wells with a nontargeting control siRNA, GFP-targeting control siRNAs, and the All Star death siRNA (Qiagen) that was used to monitor knockdown efficiency. Triplicate sets of plates were reverse transfected with 10 nM siRNA using Lipofectamine RNAiMAX transfection reagent (Invitrogen) and incubated for 72 h before treating cells with 0.6 M sorbitol for 4 h. Each plate also contained control wells in which DMEM was used instead of sorbitol. Cells were fixed and stained as described below. The images were analyzed on a Columbus Image Analysis Server (Perkin Elmer) using an embedded Acapella Image Analysis Software (Perkin Elmer) script. Nuclei were segmented and defined using their Hoechst 33342 staining. The cytoplasmic region was defined as a ring of 5 pixel width that encircled the nucleus 3 pixels away from its outside edge.^31^ The nuclear/cytoplasmic ratio of hnRNPA1 was calculated from the average intensity of hnRNPA1 fluorescent signal measured per cell within these regions. The standard Z'-factor^32^ was calculated using control siRNA-transfected cells treated with sorbitol or DMEM.

### HRV and coxsackievirus infection and hypertonic stress

For HRV infection, 4.0E05 cells in 12-well culture plates were infected by removing culture medium from cells, then rinsing cells once with PBS buffer, and adding HRV (HRV-14 generously provided by Dr. Nahum Sonenberg, Montreal, QC, Canada) or PBS (sham, negative control) to cells. Plates were then incubated at 37 °C and 5% CO_2_ for 1 h with gentle swirling every 15 min. Then, 1.0 ml of DMEM was added to cells and infection was carried out for up to 24 h as indicated. For coxsackievirus type B3 (CVB3) infection, HeLa cells were infected with CVB3 (Kandolf strain) at a multiplicity of infection (MOI) of 10 for 3 or 6 h as indicated.

For kinetic cell imaging experiments, the medium was supplemented with 1 : 5000 CellPlayer reagent (Essen Biosciences, Ann Arbor, MI, USA; see below) to detect caspase 3/7 activity. For hypertonic stress experiments, 1.2E06 cells in a 6-well culture plate were treated with DMEM containing 0.6 M (U2OS cells) or 0.4 M (HeLa T4+ cells) D-sorbitol (Sigma-Aldrich Co., Oakville, ON, Canada) or with DMEM (negative control). Cells were then placed in 37 °C and 5% CO_2_ incubator for 4 h. For kinetic cell imaging experiments the medium was supplemented with 1 : 5000 CellPlayer reagent (Essen Biosciences; see below).

### Protein extraction and western blot analysis

Cells were washed in 1 ml PBS and lysed in 50–100 *μ*l RIPA buffer (50 mM Tris-Cl, pH 7.4, 1 mM EDTA, 150 mM NaCl, 1% NP-40, 0.5% SDS, 0.5% sodium deoxycholate, 1% PMSF, 1% Leupeptin) for 15 min at 4 °C, followed by centrifugation at 12 000 × *g* for 10 min to pellet debris. Protein concentration was determined by Bio-Rad protein assay colorimetric assay kit following the manufacturer's protocol (Bio-Rad Laboratories Inc., Mississauga, ON, Canada) and equal amounts of protein extracts were separated by 10% SDS-PAGE and transferred to PVDF membrane. Samples were analyzed by western blotting using mouse anti-hnRNP A1 (Sigma, Oakville, ON, Canada), anti-HK2 (Cell Signaling Technology, Denver, CO, USA), anti-Bcl-xL (Cell Signaling Technology), anti-CVB3 VP1 (Dako, Burlington, ON, Canada), anti-*β*-actin (Abcam, Toronto, ON, Canada), anti-FLAG (Stratagene, La Jolla, CA, USA), or rabbit anti-HRV (a generous gift from Dr. Wai-Ming Lee, University of Wisconsin, Madison, WI, USA), followed by species-specific HRP-conjugated (Cell Signaling Technology) or Alexa 680-conjugated (Invitrogen) secondary antibodies. Antibody complexes were detected using an ECL, ECL Prime system (GE Biosciences, Mississauga, ON, Canada), and exposure on X-ray film or by detection using the LI-COR Odyssey infrared scanner (LI-COR Biosciences, Lincoln, NE, USA). Densitometry analyses were performed using the LI-COR Odyssey software.

### RNA extraction and qRT-PCR

Total RNA was extracted using an Absolutely RNA miniprep kit according to the manufacturer's instructions (Agilent Technologies, Mississagua, ON, Canada) and quantified using NanoDrop ND-1000 Spectrophotometer (Thermo Scientific). cDNA was generated using an oligo(dT)_18_ primer and qScript cDNA Supermix (Quanta Biosciences, Gaithersburg, MD, USA) as per the manufacturer's protocol. The synthesized cDNA was used as the template for quantitative PCR using PerfeCTa SYBR Green FastMix (Quanta Biosciences) along with gene-specific primers for HRV (forward 5′-GATCAGGTGGATTTTCCCTC-3′ reverse 5′-GTGATTGACCAGCTGATGATG-3′) and GAPDH (forward 5′-ACAGTCAGCCGCATCTTCTT-3′ reverse 5′-ACGACCAAATCCGTTGACTC-3′) and analyzed on a Mastercycler ep *realplex* (Eppendorf, Mississagua, ON, Canada) real-time PCR system using the associated realplex software. Relative expression levels were determined using the standard curve method.

### Immunofluorescence and confocal imaging

Cells were seeded onto square coverslips (22 × 22 mm, no. 1 thickness, VWR, Mississauga, ON, Canada) in 6-well culture plate as described above. Cells were rinsed 3 times with PBS, and then fixed using 3.7% formaldehyde diluted in PBS for 15 min. Cells were then rinsed once with PBS and permeabilized with 0.2% Triton-X 100-PBS buffer for 5 min followed by blocking with FBS blocking buffer (0.1% FBS, 0.2% BSA, 0.004% Triton-X 100, diluted in PBS buffer) for 15 min. Primary antibody (anti-hnRNP A1 (1 : 300) or anti-HK2 (1 : 500) dilution in Triton-X 100/BSA buffer: 0.2% BSA, 0.004% Triton-X 100, diluted in PBS buffer) was added for 1 h, followed by 3 washes for 5 min each with Triton-X 100/BSA buffer. Secondary antibody (Alexa Fluor anti-mouse 488 or anti-rabbit 594, 1 : 1000 dilution in Triton-X 100/BSA buffer) was added to coverslips for 1 h. Cells were washed 3 times for 5 min each with Triton-X 100/BSA buffer. Nuclei were stained with Hoechst 33342 solution (trihydrochloride trihydrate, 1 *μ*g/ml, Invitrogen) diluted in PBS for 5 min. Finally, cells were washed 3 times for 5 min each time with PBS, and mounted onto glass slides using Dako Fluorescent Mounting Medium (Dako Inc., Burlington, ON, Canada). Cells were imaged using the Olympus Fluoview FV-1000 Laser Confocal Microscope (Toronto, ON, Canada) and software with × 20, × 40, or × 60 oil-immersion objectives, as indicated.

### Kinetic cell imaging

Cells were seeded in 6-well or 12-well culture plates in the presence of CellPlayer Kinetic Caspase-3/7 Apoptosis Assay Reagent (1 : 5000; Essen Biosciences) and imaged at 1 h intervals for 24 h using the IncuCyte Zoom Live Content Kinetic Imaging System (Essen Biosciences). Activated caspase 3/7 fluorescence and cell confluency over time was measured using the IncuCyte Zoom software and data were analyzed in GraphPad Prism 5.00 (San Diego, CA, USA).

### Statistical analysis

An unpaired *t*-test was performed using GraphPad Prism version 5.00 for Windows (GraphPad Software) to determine *P*-value in repeated experiments. All results are shown as mean±S.D. For confocal microscopy, data were collected from images by cell counting in multiple randomized fields by blinded observer. For kinetic cell imaging experiments, data were collected using IncuCyte Zoom software. For RT-qPCR experiments, average RNA expression was calculated using data collected from three biological replicates and three technical replicates for each biological replicate. Unless otherwise noted, all results were obtained through a minimum of three independent experimental replications.

## Abbreviations

HK2: hexokinase 2
hnRNP A1: heterogeneous nuclear ribonucleoprotein A1
HRV: human rhinovirus
MAPK: mitogen-activated protein kinase
XIAP: X chromosome-linked IAP
Bcl-xL: B-cell lymphoma-extra large
FGF-2: fibroblast growth factor-2
S6K2: ribosomal protein S6 kinase 2
eIF2*α*: eukaryotic initiation factor 2 *α* subunit
3-BrPa: 3-bromopyruvate
mTOR: mechanistic target of rapamycin
IRES: internal ribosome entry site
