# Supplementary material for: Hexokinase 2 controls cellular stress response through localization of an RNA-binding protein
Source: Cell Death Dis. 2015 Aug 6;6(8):e1837–. doi: 10.1038/cddis.2015.209 (PMC4558502; doi:10.1038/cddis.2015.209)
Supplement: Supplementary Information [file cddis2015209x1.pdf]

**Supplemental Figure S1. Hexokinase 2 is required for cytoplasmic accumulation of hnRNP A1 during hypertonic stress.** HeLa T4+ cells were transfected with HK2-targeting (siHK2) or non-targeting control (siCTRL) siRNAs for 48 hours. Representative confocal microscopy images of hnRNP A1 localization and HK2 abundance by immunofluorescence in DMEM or sorbitol treated cells are shown. The western blot analysis of imaged cells is shown in Figure 2A.

**Supplemental Figure S2. Ectopic Overexpression of Hexokinase 2 has no appreciable effect on hnRNP A1 abundance and localization.** HeLa T4+ cells were transiently transfected with HK2-expressing (FLAG-HK2) or control (EV) plasmid for 48 hours and subsequently analysed for the abundance (by western blotting, **(A)**) or subcellular localization (by immunofluorescence, **(B)**) of hnRNP A1.

**Supplemental Figure S3. Coxsackievirus type B3 (CVB3)-induced cytoplasmic accumulation of hnRNP A1 is independent of hexokinase 2.** HeLa cells were transfected with HK2-targeting (siHK2) or non-targeting control (siCTRL) siRNAs followed by sham (control) or CVB3 infection for 3 or 6 hours as indicated. **(A)** Expression of hnRNPA1 (left panel) and CVB3 capsid protein VP1 (right panel) was visualized by immunofluorescence. **(B)** Protein levels of HK2, hnRNP A1, VP1, and β-actin (loading control) were examined by western blot analysis. MW marker is indicated on the left.

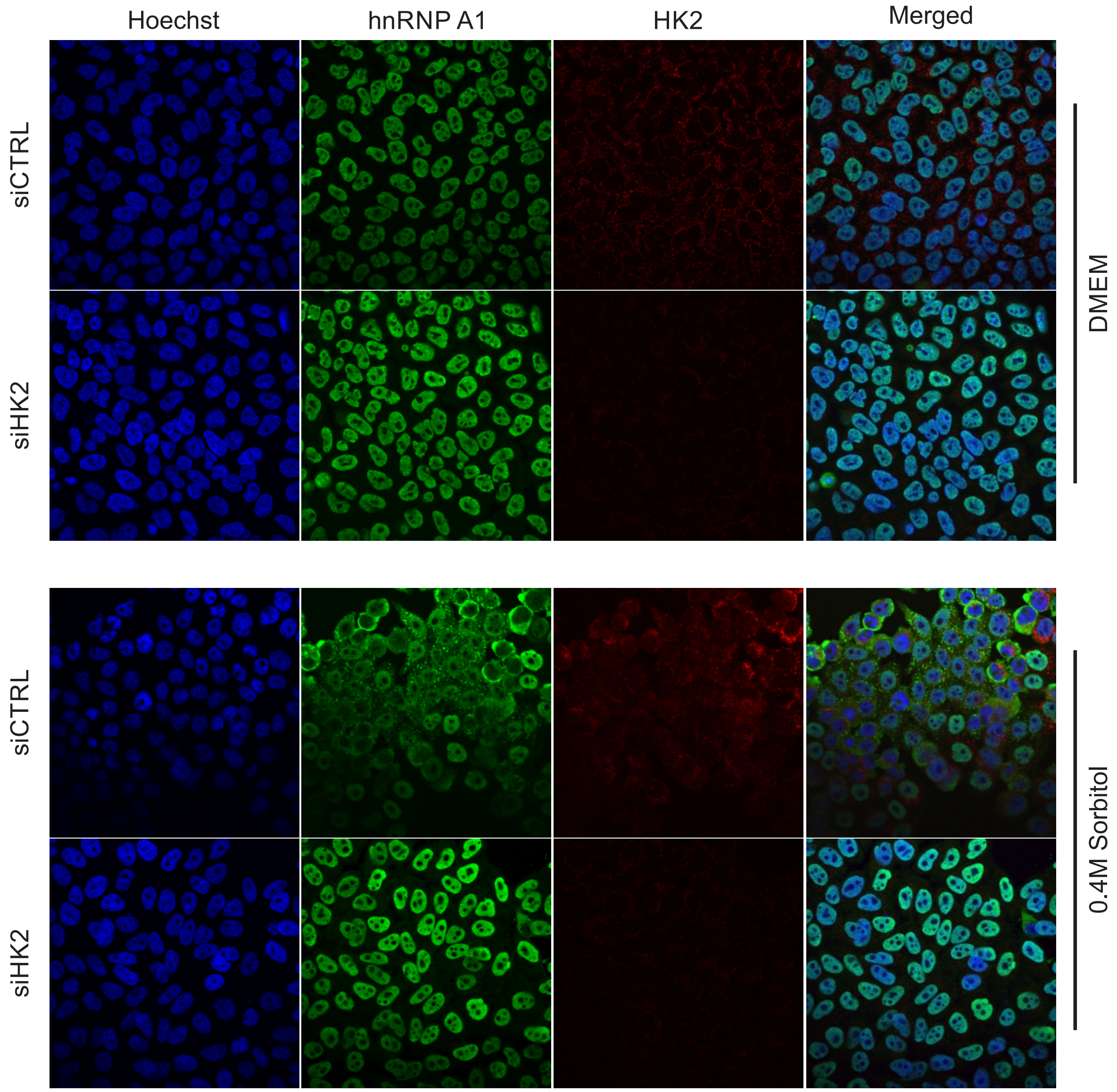
Hoechst
hnRNP A1
HK2
Merged
siCTRL
siHK2
DMEM
siCTRL
siHK2
0.4M Sorbitol

**A.**

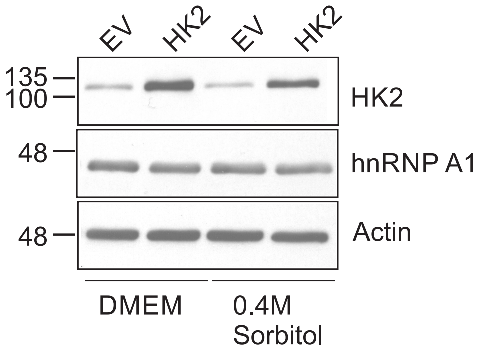
EV
HK2
EV
HK2
135
100
HK2
48
hnRNP A1
48
Actin
DMEM
0.4M Sorbitol

**B.**

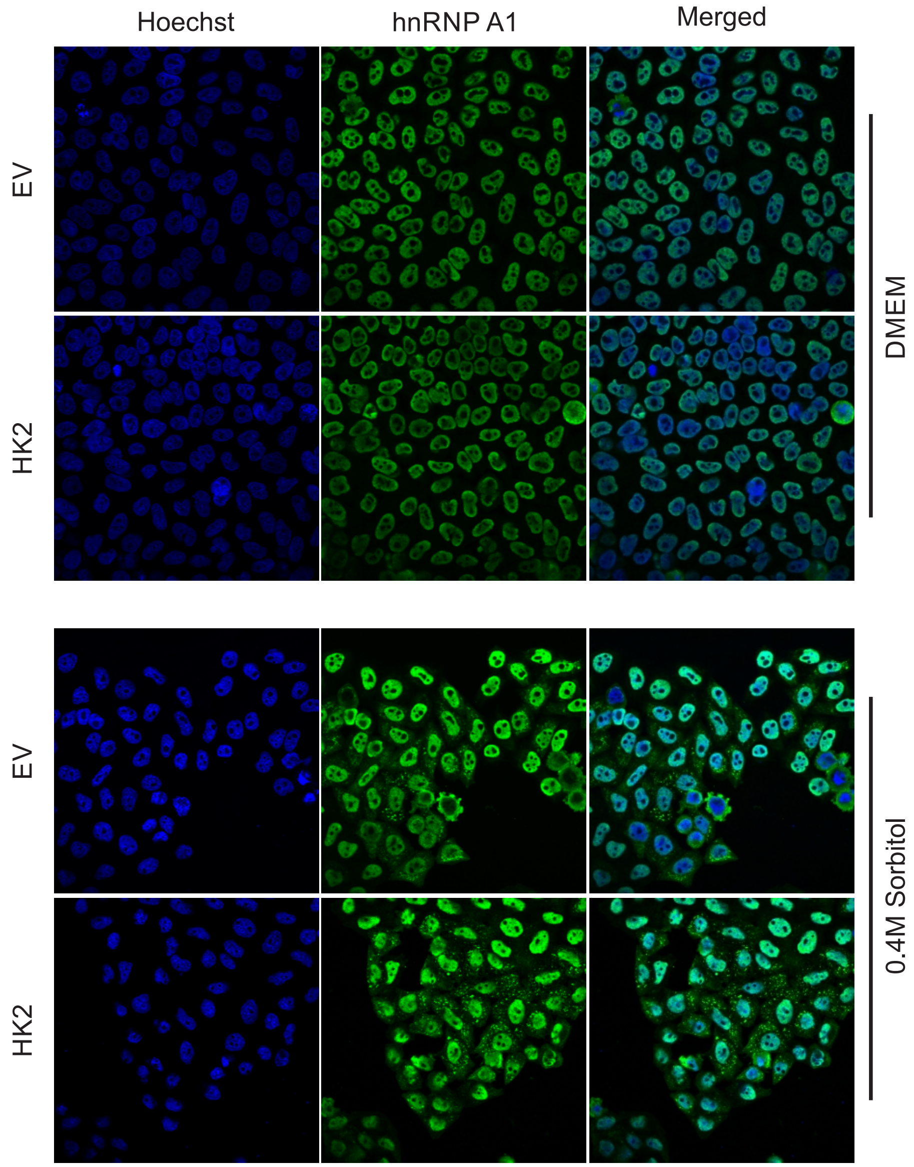
Hoechst
hnRNP A1
Merged
EV
HK2
DMEM
EV
HK2
0.4M Sorbitol

**A.**

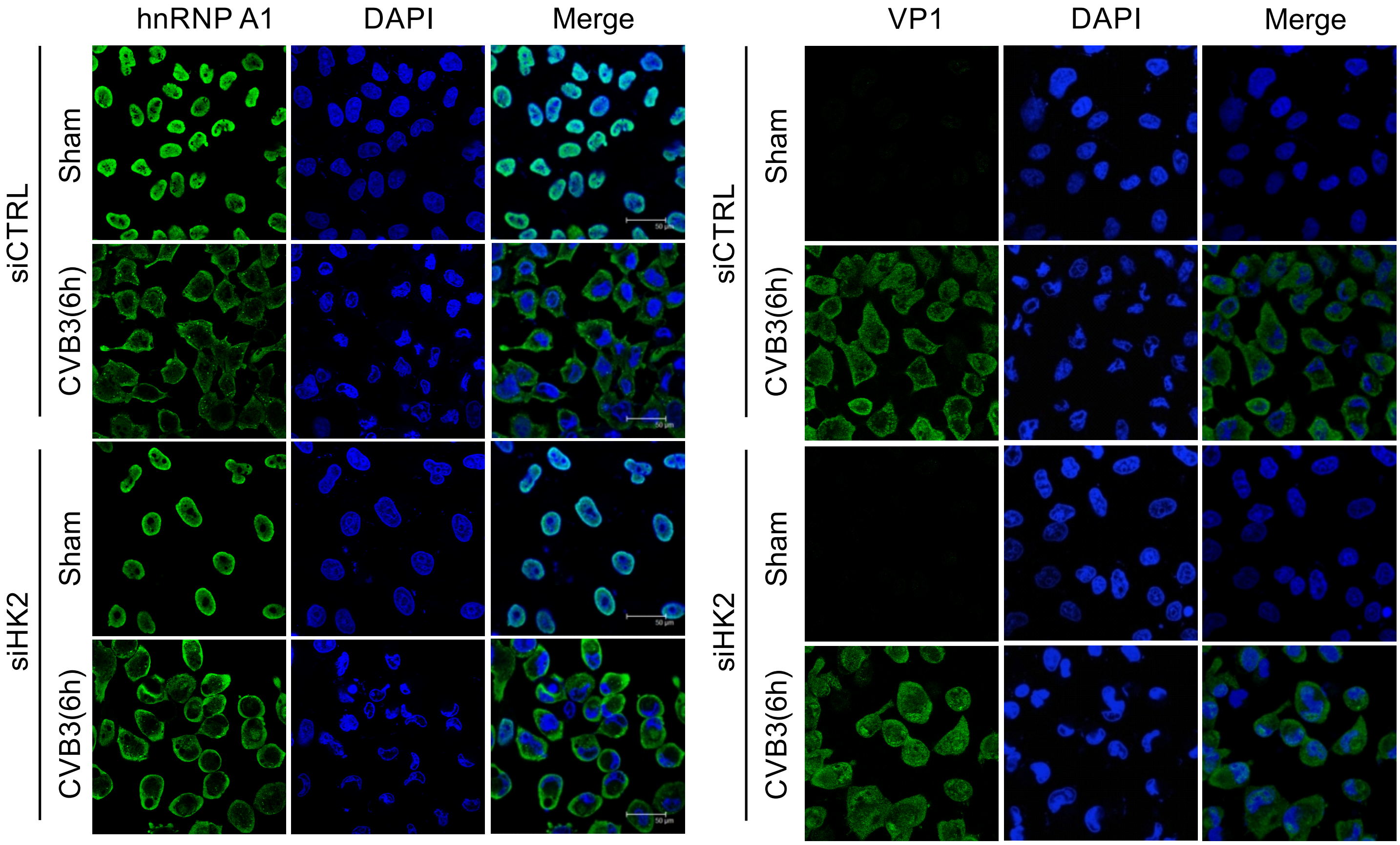
hnRNP A1
DAPI
Merge
VP1
DAPI
Merge
siCTRL
siHK2
Sham
CVB3(6h)
Sham
CVB3(6h)
siCTRL
siHK2
Sham
CVB3(6h)
Sham
CVB3(6h)

**B.**

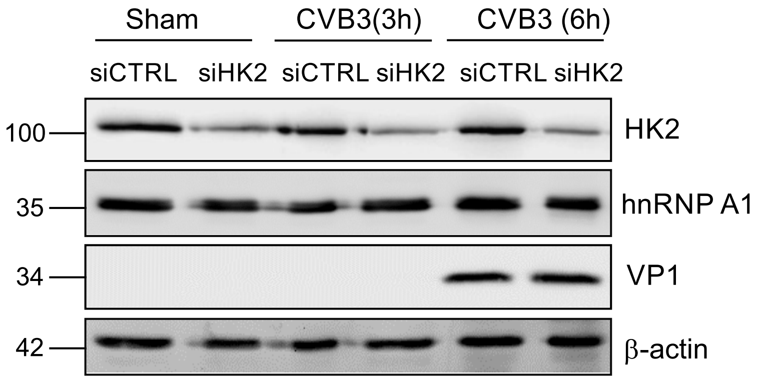
Sham
CVB3(3h)
CVB3 (6h)
siCTRL siHK2 siCTRL siHK2 siCTRL siHK2
100
35
34
42
HK2
hnRNP A1
VP1
β-actin
